# Serious Games: A new Approach to Foster Information and Practices About Covid-19?

**DOI:** 10.3389/frobt.2022.830950

**Published:** 2022-05-23

**Authors:** L. Montalbano, L. Gallo, G. Ferrante, V. Malizia, G. Cilluffo, S. Fasola, M. Alesi, S. La Grutta

**Affiliations:** ^1^ Institute for Biomedical Research and Innovation (IRIB), National Research Council of Italy, Palermo, Italy; ^2^ Institute for High Performance Computing and Networking (ICAR), National Research Council of Italy, Napoli, Italy; ^3^ Department of Surgical Sciences, Dentistry, Gynecology and Pediatrics, Pediatric Division, University of Verona, Verona, Italy; ^4^ Department of Psychology, Educational Science and Human Movement (SPPEFF), University of Palermo, Palermo, Italy

**Keywords:** serious game, COVID-19, information, knowledge, practices

## Abstract

The current Covid-19 pandemic poses an unprecedented global challenge in the field of education and training. As we have seen, the lack of proper information about the virus and its transmission has forced the general population and healthcare workers to rapidly acquire knowledge and learn new practices. Clearly, a well-informed population is more likely to adopt the correct precautionary measures, thus reducing the transmission of the infection; likewise, properly educated healthcare workers are better equipped to manage the emergency. However, the need to maintain physical distancing has made it impossible to provide in-presence information and training. In this regard, new technologies have proved to be an invaluable resource by facilitating distance learning. Indeed, e-learning offers significant advantages because it does not require the physical presence of learners and teachers. This innovative method applied to serious games has been considered potentially effective in enabling rapid and large-scale dissemination of information and learning through content interactivity. We will review studies that have observed the development and use of serious games to foster information and practices about Covid-19 aimed at promoting behavioral changes in the population and the healthcare personnel involved on the front line.

## Introduction

In the pandemic context we are going through, education and training represent strategic elements to meet the needs of a population that was unprepared to face an unprecedented emergency situation. Therefore, it is necessary to respond in a very short period of time to global needs that bear multi-professional and multidisciplinary implications, and to manage the uncertainty in content and procedures, in the context of a reorganization of the existing educational system, consolidating internal and external communication based on increasingly effective criteria (https://www.who.int/westernpacific/about/how-we-work/programmes/who-health-emergencies-programme). All these elements have determined a unique situation not only from a health and social point of view, but also in terms of staff training with the need and urgency to offer real-time and adequate training to the widest possible population. This context, however, has generated the need to develop knowledge and skills to deal with emergencies outside the common classroom setting. Moreover, the type of training required cannot only be aimed at transferring information, but also at adopting preventive and protective behaviors for large-scale change, both by healthcare personnel and, indirectly, by the population (Lin et al., 2014).

Indeed, the inadequate application of Infection Prevention and Control (IPC) procedures by the population and, in particular, by healthcare personnel, and the improper use of personal protective equipment (PPE) can facilitate the spread of Covid-19 infection (Casanova et al., 2008; Zhang et al., 2020; Barycka et al., 2021). Therefore, building and strengthening the intrinsic motivation of the population and health care personnel to strictly apply the IPC guidelines and do their best to avoid the continuation of the pandemic is an indispensable goal to be achieved (Handbook of self-determination research., 2002). The need for mass training, with special regards to respecting physical distancing, required the creation of original and challenging material to promote guidelines.

Serious games have motivational properties that can enhance engagement and satisfaction by conveying important messages (Gentry et al., 2019). A serious game is an educational tool that, through an engaging and self-reinforcing scenario, manages to motivate and educate the player; it requires the ability to solve a problem using knowledge, skills, initiative, and strategy. Competition, rewards and fun are the principles of gamification used in various educational contexts to promote player enjoyment and retention of acquired knowledge (Evans et al., 2015). In recent years, serious games have been used and recognized as a potential tool to improve education in health care settings as well (Olszewski and Wolbrink, 2017; Blanié et al., 2020). Of note, the use of serious games in vulnerable populations during the COVID-19 pandemic has been recently considered as a promising tool in enhancing physical activity and healthy movement behaviors (Ferrante et al., 2021; Malizia et al., 2021). In such a peculiar situation as the COVID-19 pandemic, when online education and training seem to be the only alternative to reach as wide and heterogeneous an audience as possible, these games could be very effective.

This mini-review is intended to provide an overview of studies that have shown the development and use of serious games to foster information and practices on Covid-19 aimed at promoting behavioral changes in the population and the frontline health-care personnel.

## Methods

On October 26, 2021, we searched PubMed, Scopus and Web of Science, using the search string “serious game*” AND “Covid-19”. The inclusion criteria were: 1) English language; 2) Serious games developed and used to foster information and practices on Covid-19. The exclusion criteria were: 1) off-topic; 2) case reports, reviews, or conference proceedings. The studies identified in the three databases were combined, and duplicates were removed. Two reviewers (LM and SF) screened the articles in terms of relevance based on titles and abstracts. The same reviewers screened the full texts of potentially eligible articles. The following information was extracted from the included articles: first author and publication year, study design (including sample size and age), country, study aim, type of serious game, outcome assessment and main results.

## Results

**FIGURE 1:**
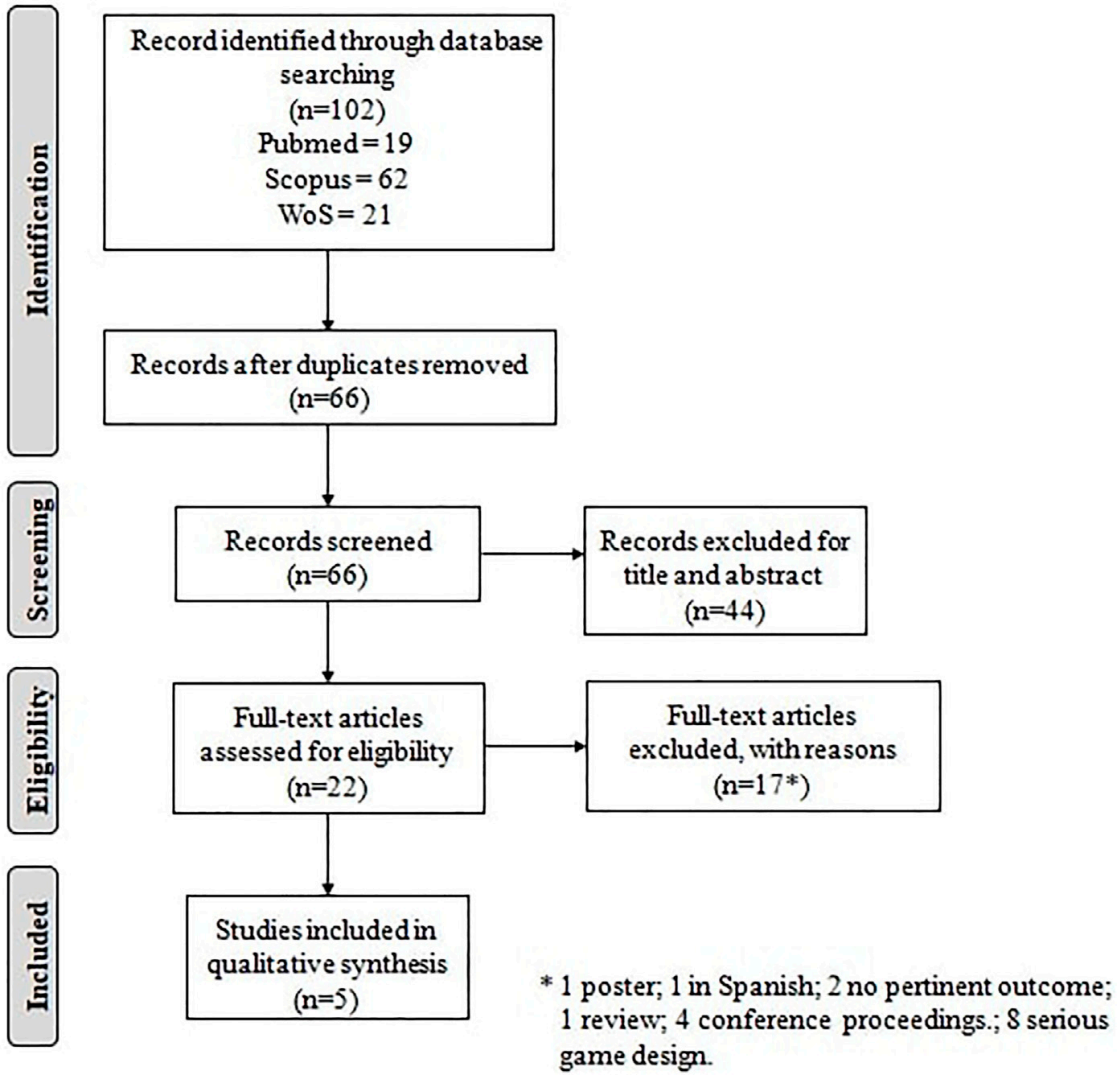
Flow diagram showing the study selection process.

Figure 1 summarizes the study selection process. We identified 102 articles across the three electronic databases. After excluding any duplicates, 66 articles were screened based on titles and abstracts, and 22 articles were identified as potentially eligible. Among these, 17 were excluded following full-text evaluation. Therefore, 5 articles were included in this review. Table 1 summarizes the characteristics of the studies included. We identified four serious game designed to promote knowledge and teach new practices with regard to Covid-19: 1) Escape COVID-19 teaches COVID-19 IPC practices (Suppan et al., 2021b; 2021a); 2) Point of Contact (PoC) explains COVID-19 preventive measures (Hill et al., 2021); 3) Covidgame is about COVID-19 education for students attending the last year of medical school (Hu et al., 2021); 4) Plague Inc. aims at providing knowledge about Covid-19 transmission and characteristics of pathogens (Peng and Bai, 2021). The studies included were carried out in different countries and targeted different populations (general population, healthcare personnel and young adults).

**TABLE 1:** Studies included.

Firts author and year	Study design	Participants	Country	Aim	Serious game	Outcome assessment	Results
Peng and Bai 2021	Cross-sectional study	General population (≥ 20 years)	China	Investigate whether the gaming experience indirectly influenced the public's knowledge, attitudes, and practices (KAP) regarding COVID-19.	*Plague Inc.* A serious game designed to introduce the public to epidemiology in an unconventional way, allowing users to obtain knowledge of the transmission and characteristics of pathogens through gaming.	A online survey consisted of four parts: demographics, KAPof COVID-19, game experience of Plague Inc., and a gameful experience scale.	Serious games highlighting the theme of pathogen transmission may enhance public knowledge and attitudesregarding COVID-19. Furthermore, the creative thinking and dominance involved in gameful experiences may act as criticalfactors in public attitudes and practices regarding COVID-19.
Suppan et al. 2021	Prospective web-basedstudy	General population (average of 40 years)	Switzerland	Identify the factors facilitating or impeding the intention of changing Infection Prevention and Control (IPC) behavior after following this game in a larger and more heterogeneous population after national rollout.	*Escape COVID-19* A serious game designed to teach COVID-19 IPC practices.	A pre-questionnaire designed to gather demographic data and to assess the initial level of knowledge regarding SARS-CoV-2 transmission and IPC guidelines.A post-questionnaire designed to assess whether the participants intend to change their IPC practices after completing the game.	“Escape COVID-19” is a useful tool to enhance correct IPC measures on a national scale, even after 2 COVID-19 pandemic waves.
Suppan et al., 2021	Web-Based Randomized Controlled Trial	Employees of Long-term Care Facilities	Switzerland	Determine whether LTCF employees were willing to change their IPC practices after playing “*Escape COVID-19*.”	*Escape COVID-19* A serious game designed to teach COVID-19 IPC practices.	A pre-questionnaire designed to gather demographic data and to assess the initial level of knowledge regarding SARS-CoV-2 transmission and IPC guidelines.A post-questionnaire designed to assess whether the participants intend to change their IPC practices after completing the game.	The serious game “Escape COVID-19” was more successful than standard IPC material in convincing Long-Term Care Facilities (LTCF) employees to adopt COVID-19–safe IPC behavior.
Hill et al. 2021	The study was a mixed methodsstudy relying both on questionnaires and quantitative data from game logs.	Students of Lancaster University (19–25 years)	United Kingdom	Investigate effects of a serious game on COVID-19 perception and behavior.	*Point of Contact (PoC)* A multiplayer serious game for COVID-19 preventive measures.	A pre-questionnaire aimed at assessing the participants’ knowledge of COVID-19 preventive measures before entering the study. A post-questionnaire to measure any changes in the perceived importance of the preventive measures.	The results show a significant positive change to participants’ perceptions towards COVID-19 preventive measures, shifting perceptions towards following guidelines more strictly dueto a greater awareness of how the virus spreads.
Hu et al. 2021	Case-control study	Final year medical students (average of 22 years)	China	Explore the effectiveness of *Covidgame*, compared with an online lecture.	*Covidgame* A serious game designed for the COVID-19 education of the final year medical students.	All medical students were tested on three separate occasions: before, immediately after, and 5 weeks after the lesson. Each test contained 10 multiple-choice questions (one point per item) that assessed the students’ knowledge of COVID-19.	This effective learning mode allowed students to master information related to COVID-19 in the short and long term.

### Studies Included

#### Serious Games for General Population

Confronted with such a severe threat as COVID-19, a population with higher levels of knowledge on the issue is considered more likely to take appropriate preventive measures and thus reduce the prevalence rate of the infection (Aburto et al., 2010; Lin et al., 2014). Indeed, many countries in the world have proactively provided up-to-date COVID-19 information and prevention guidelines to citizens using a variety of media (Mheidly and Fares, 2020). Among them, serious games have been used to promote public attention to health, hygiene and medical care, making it possible to understand any health threats and medical challenges in such a difficult global situation with reference to health and safety (Brox et al., 2011; Devlin et al., 2014; Robinson et al., 2018). Teaching medical knowledge through educational games has been found to be as effective as traditional teaching methods (Crovato et al., 2016; Abraham et al., 2019; Tubelo et al., 2019). Therefore, to spread COVID-19 prevention messages in an original and engaging way, some serious games have been developed, such as Plague Inc. and Escape COVID-19, which have received considerable attention during the pandemic (Suppan et al., 2021b; Peng and Bai, 2021).

*Plague Inc.* is a serious game designed to introduce the public to epidemiology in an unconventional way, allowing users to be informed of the transmission and characteristics of pathogens through gaming. Peng and Bai used a cross-sectional study design to explore the impact of Taiwanese gameful experiences on public knowledge, attitudes, and practices (KAP) regarding COVID-19, with the aim to investigate the effects of gameful experience of Plague Inc. on public KAP regarding COVID-19 by means of online surveys (Peng and Bai, 2021). A total of 486 participants were recruited for this study, 200 women and 286 men, mostly aged between 20 and 29, who were living with others, had a university degree and a job. In total, 276 participants indicated that they had played Plague Inc. while 210 that they had never played the game. An online survey was used to assess demographics, KAP of COVID-19, game experience of PlagueInc., and a gameful experience scale. Results showed that participants who had played Plague Inc. demonstrated higher levels of knowledge (*p* = 0.03, median 7, IQR 7–8) and attitudes (*p* = 0.007, median 8, IQR 7–8) than participants who had not played Plague Inc. (knowledge: median 7, IQR 6–8; attitude: median 7, IQR 6–8) and a significant correlation between creative thinking (*ρ* = 0.127, *p* = 0.04) and dominance (*ρ* = 0.122, *p* = 0.04) in attitude and between creative thinking (*ρ* = 0.126, *p* <.001) and dominance (*ρ* = 0.119, *p* = 0.049) in practice. The study demonstrated that Plague Inc., by highlighting the topic of pathogen transmission, may enhance public knowledge and attitudes regarding COVID-19.

Another serious game that achieved success during the pandemic was *Escape COVID-19*, a serious game designed to improve knowledge and application of IPC procedures, based on Nicholson's concept of meaningful gamification that enhances player engagement and helps fostering knowledge increasing satisfaction, and developing skills, which could contribute to the promotion and implementation of appropriate behaviors. Suppan et al. conducted a fully-automated prospective web-based study with the aim of identifying the factors facilitating or impeding the intention of changing IPC behaviors after playing this game in a larger and more heterogeneous population, in the three main linguistic regions of Switzerland (Suppan et al., 2021b). Participants completed a short demographic questionnaire before starting the serious game and a second questionnaire had to be completed at the end of the game in order to obtain a certificate of completion that participant would have received as an incentive. The primary outcome was the proportion of participants reporting to be willing to change their IPC behaviors; secondary outcomes were the IPC areas affected by this willingness and the presumed evolution in the use of specific personal protective equipment items, also assessing items associated with the intention to change or not to change IPC behaviors; further secondary outcomes were subjective perceptions regarding the length, difficulty, meaningfulness, and usefulness of the game, the impression of engagement or boredom during the game, and the willingness to recommend it to friends or colleagues. 1104 participants (34.2%) completed the game and post-game questionnaire; among these, 509 (46.1%) responded that they intended to change their IPC behaviors after playing; among the others, most (86%, 512/595) responded that they were already applying these guidelines. However, participants who followed the German version were less likely to change their IPC behaviors (OR 0.48 [95%CI 0.24 to 0.96], *p* = 0.038) and found the game less engaging (*p* <.001). Participants aged 53 years or older were more likely to change IPC behaviors (OR 2.07 [95%CI 1.44 to 2.97], *p* <.001). Results showed that Escape COVID-19 is a useful tool to improve IPC-adjusted measures on a national scale, following the COVID-19 pandemic. However, its impact was influenced by the language and age of participants, and their educational background. Therefore, some adaptations are needed.

### Serious Games for Healthcare Personnel

Nursing home residents are among people at higher risk of complications and death in case they develop symptoms of COVID-19 (Burki, 2020; O’Driscoll et al., 2021). In these facilities, the virus can be carried by even a single employee, who can potentially foster rapid transmission between residents and colleagues (Arons et al., 2020; McMichael et al., 2020). In nursing homes, as in any other place, viral transmission is often facilitated by poor adherence to IPC guidelines, as observed by a recent systematic review that identified the need to promote hygiene and the use of appropriate personal protective equipment to prevent virus transmission (Rios et al., 2020; McDonald et al., 2021). However, the physical and social distancing imposed by the pandemic may hinder the dissemination of these guidelines, just as their application may be compromised by contradictory messages or lack of trust in the guidelines provided (Houghton et al., 2020). In this sense, it is necessary to act on health workers’ motivation in adopting good IPC practices and gamification, as a new paradigm for engagement and empowerment that could help the effective dissemination of guidelines and the promotion of safe practices against COVID-19, promoting the player’s engagement to foster knowledge, satisfaction and skills (Nicholson, 2015). *Escape COVID-19* was developed by Suppan et al. to promote IPC practices with a specific focus on COVID-19 among HCWs and other hospitals’ employees (Suppan et al., 2020a). A web-based randomized controlled trial has been carried out on nursing home employees with the aim of improving knowledge and application of IPC procedures, with the intention to change their IPC practices (Suppan et al., 2021a). The primary study outcome was the proportion of participants willing to change their IPC practices; secondary outcomes included the analysis of specific questions detailing the exact changes considered by the participants. Participants were selected from all nursing home employees working in Geneva to represent a convenience sample, independently on their occupational status or the potential specificities of the facilities where they worked, with no exclusion criteria, and an estimated number of eligible employees of about 4000 were estimated with a required participation rate of around 20%. Participants were randomized to either the control or the serious game (intervention) group and each of them completed a questionnaire before and after playing. The first questionnaire was designed to gather demographic data and to assess the initial level of knowledge regarding SARS-CoV-2 transmission and IPC guidelines; the second questionnaire was designed to assess whether participants intend to change their IPC practices. A total of 295 sets of answers were analyzed and the serious game Escape COVID-19 proved to be more successful than standard IPC material in convincing Long-Term Care Facilities (LTCF) employees to adopt COVID-19–safe IPC behavior.

### Serious Games for Young Adults

The WHO identifies young adults to be the least compliant to preventive measures, because the information provided by various communication channels often uses a language that is difficult for young people to understand, with the result of lowering their risk perception as well as their compliance with preventive measures (Buckingham, 1997; https://www.reuters.com/article/uk-healthcoronavirus-who/who-message-to-youth-on-coronavirus-you-are-not-invincible-dUKKBN217347, 217347). In this sense, the challenge is the ability to transform complex information about COVID-19 into perception-modifying knowledge, through learning from experience (Bodner, 1986). In the specific case of COVID-19, a safe virtual tool that recreates the experience of how infection transmission occurs, for example, could help transfer the virtual experience to the real world, as already demonstrated for other health issues (Beale et al., 2007; Ortiz de Gortari and Griffiths, 2012; Luz et al., 2016).

In this regard, a study by Hill et al. described the development and evaluation of a multiplayer serious game, *Point of Contact* (PoC), designed to improve young gamer’' perceptions of preventive measures against COVID-19. Twenty-three participants (16 males, 7 females) with significant gaming experience and aged between 19 and 25 years were recruited from Lancaster University (Hill et al., 2021). Students’ knowledge about COVID-19 preventive measures was assessed through filling out a pre-study questionnaire; the same questionnaire was completed at the end of the study to measure any change in their perceived importance of preventive measures. Additional questions were included in the post-study questionnaire to identify whether the player’s actions matched real-world behavior. Study results showed satisfaction by the participants, who felt they were well-informed about virus transmissibility, and learned what preventive measures to implement and expressed their intention to follow them. In addition, the PoC increased not only their positive perception of preventive measures, but also their acceptance of an approach that prioritizes health over money. Therefore, the use of a serious game had an effect on participants who reported following guidelines more strictly, using personal protective equipment more frequently, and prioritizing safety over money.

Medical students in particular need to learn about the virus because they are likely to deal with COVID-19 patients after graduation (Choi et al., 2020). Therefore, for this specific target population, a study conducted by Hu et al. aimed at evaluating the effectiveness of a serious game versus online classes in improving knowledge about COVID-19, as in recent years serious games have been recognized as a potential tool to improve medical education (Hu et al., 2021). The study involved the development of a 3-h lecture on COVID-19, containing notions of epidemiology, identification and clinical manifestations, laboratory findings, diagnosis, and evaluation of COVID-19 patients, as well as use of PPE; the lesson was then taught to students attending the final year of medical school. The *Covidgame* serious game was concurrently designed and developed to assist students in mastering the topic. Education was provided to participants (*n* = 129), divided into two groups, as follows: for the first group (*n* = 60), in the form of an online lecture; for the second group (*n* = 69), in the form of an educational game. Finally, to assess the effect of these educational methods, all teachers were asked to administer three online tests to each student: a pre-lesson test, a post-lesson test, and a final online test 5 weeks after the lesson. The results showed no significant differences between the two methods in terms of short-term knowledge increase; however, the group of students who had used the serious game scored significantly higher in knowledge retention than the online lecture group (*p* = 0.001). Therefore, during a time of disruption in traditional university teaching due to the COVID-19 pandemic, Covidgame proved to be a potentially effective tool for the long-term medical education of students.

## Discussion

Considering the unexpected and unprecedented emergency the entire world had to face, the core health strategies have focused on allowing easy access to information and guidelines aimed at preventing the spread of the COVID-19 disease. The WHO has taken advantage of its global reach and various communication channels to disseminate indications on how to prevent or delay the transmission of the disease, and recommendations on personal hygiene, social distancing and the appropriate use of face masks. However, for these measures to be truly effective, community awareness and a strong commitment towards the control of the disease are necessary (WHO/COVID-19/Community_Transmission/2020.1). Only a well-informed population can make conscious decisions and adopt positive behaviors to protect themselves and others (Aburto et al., 2010; Lin et al., 2014), given that poor health literacy has been associated to poor compliance with protective behaviors (Castro-Sánchez et al., 2016). These are modulated by risk perception that is largely based on the information received. Therefore, it is crucial that communication be clear and easy to avoid negative emotional reactions, cognitive distortions, and erroneous assessments and decisions that could hamper the adoption of proper behaviors (Glik, 2007).

Digital technologies have contributed to the quick, clear and easy dissemination of information on COVID-19 in order to promote changes in the behavior of the population (Bai et al., 2020). In particular, literature has observed how serious games can provide significant advantages for the dissemination of information and learning by increasing user’s interactivity and providing wide information coverage, though not his/her physical presence (Suppan et al., 2020b).

Indeed, a serious game is developed not with the main purpose of entertaining and fun (Djaouti et al., 2011), but as a powerful tool for the development and acquisition of new knowledge and skills by experienced users as well as beginners (Caserman et al., 2020). Despite the limited number of studies on the development and application of serious games for promoting knowledge, attitudes and practices on the new Coronavirus, the available results seem to be really encouraging.

Among the general population, older participants in the *Escape COVID-19* game had reported that they were more likely to change their virus-prevention behaviors after playing, possibly because of the association between their older age and a higher risk of complications from COVID-19 or due to the often-unclear informational material (Levin et al., 2020; Suppan et al., 2021b). Therefore, an engaging and clear way of providing information might be appropriate to convey critical messages to this target population. Indeed, contrary to what one might think about games being only for younger people (Marston, 2012), literature has observed that in Generation X (people born between 1965 and 1979) e-learning processes are closely related to a stronger level of contextualization of the online content learned (Yawson and Yamoah, 2020). For young adults, given their ability to use new technologies, the introduction of serious games as an educational tool has certainly been a winning approach, as is the case of *Plague Inc.*, in which the majority of players were between 20 and 39 years old and had previous gaming experience. In comparison with older participants, during their gaming experience the younger ones showed not only higher levels of knowledge and attitudes but, most importantly, creative thinking and dominance, which are among the most significant factors influencing learning performance (Parra-González et al., 2020; Peng and Bai, 2021).

Very positive results were also observed with respect to healthcare personnel. In this case, the realistic game experience has allowed them to learn and, above all, to put into practice the acquired knowledge; this was made possible through the social involvement making the players take decisions that, in real life, would influence other people and also through the increasing level of difficulty that stimulated their interest in the game. In this way, players had the feeling to play an important role in the common effort against the pandemic and this could have been one of the main factors leading to the adoption of correct behaviors (Suppan et al., 2021a).

Other interesting results have come from the use of serious games developed among university students. In this population, which is often uninterested in health education, the game experience provided an innovative approach to learning COVID-19 safety measures in an accessible and engaging way, as is the case of *PoC*, a multiplayer serious game that increased risk awareness and compliance with virus prevention measures (Hill et al., 2021) *Covidgame* was equally valid for medical students who were forced to interrupt their in-person classes and for whom knowledge and practices against the virus represent the foundation of their future profession (Hu et al., 2021). The study suggested that a game-based computer application which meets curricular objectives is an effective learning methodology. Again, the benefits of the learning strategies were active participation and interaction in the gaming experience, as demonstrated by other studies which observed that, in teaching medical students, serious games can be easily compared to lectures in terms of knowledge acquired when assessed immediately after the application of the learning method (Rondon et al., 2013; Marcus et al., 2019).

## Conclusion

Despite due to the time scale of COVID, few papers were found and they are not necessarily of very high quality, we can state that digital health solution ad serious games can be considered a promising approach to limit the spread of Covid-19 by effectively supporting institutions and facilitating wide dissemination of information. Communication during a pandemic must quickly reach a heterogeneous audience, provide clear and objective information, and fight misinformation and fake news at the origin, thus preventing confusion and skepticism, which can negatively impact individuals and the whole society.
